# The circadian clock uses different environmental time cues to synchronize emergence and locomotion of the solitary bee *Osmia bicornis*

**DOI:** 10.1038/s41598-019-54111-3

**Published:** 2019-11-28

**Authors:** Katharina Beer, Mariela Schenk, Charlotte Helfrich-Förster, Andrea Holzschuh

**Affiliations:** 1 Department of Neurobiology and Genetics, Biocenter, University of Würzburg, Am Hubland, 97074 Würzburg Germany; 2 Department of Animal Ecology and Tropical Biology, Biocenter, University of Würzburg, Am Hubland, 97074 Würzburg Germany

**Keywords:** Behavioural ecology, Evolutionary developmental biology

## Abstract

Life on earth adapted to the daily reoccurring changes in environment by evolving an endogenous circadian clock. Although the circadian clock has a crucial impact on survival and behavior of solitary bees, many aspects of solitary bee clock mechanisms remain unknown. Our study is the first to show that the circadian clock governs emergence in *Osmia bicornis*, a bee species which overwinters as adult inside its cocoon. Therefore, its eclosion from the pupal case is separated by an interjacent diapause from its emergence in spring. We show that this bee species synchronizes its emergence to the morning. The daily rhythms of emergence are triggered by temperature cycles but not by light cycles. In contrast to this, the bee’s daily rhythms in locomotion are synchronized by light cycles. Thus, we show that the circadian clock of *O. bicornis* is set by either temperature or light, depending on what activity is timed. Light is a valuable cue for setting the circadian clock when bees have left the nest. However, for pre-emerged bees, temperature is the most important cue, which may represent an evolutionary adaptation of the circadian system to the cavity-nesting life style of *O. bicornis*.

## Introduction

Although we find evidence for circadian clocks everywhere in nature, many aspects of the underlying clock mechanisms, and the behaviors that result from them, are yet to be understood. Bees, as many other organisms, evolved an endogenous circadian clock in order to cope with the daily reoccurring environmental changes. The circadian clock enables bees to prepare for activity before the day comes, to avoid putative competitors and to synchronize with mating partners or high nectar availability that varies throughout the day^1,2^. The right timing of activities may improve survival and reproductive success of the bees. One of the circadian clock mechanisms that remain to be understood is the control of emergence of bees timed to a certain time of the day. A daily rhythm of emergence develops when the circadian clocks of bees in a population are synchronized to the 24-hour day and the clocks of all bees are set to the same time. The daily rhythm of emergence should persist in a bee population over a period of several weeks because not all bees emerge at the same date of the year (a certain variation exists within the population)^3^.

Circadian clocks are synchronized by environmental time cues, so called Zeitgebers. Examples of Zeitgebers are daily oscillations in levels of environmental factors like temperature and light or daily reoccurring feeding times and social interactions^4–6^. It is conceivable that synchronization of bee emergence also arise in the absence of a circadian clock, triggered by a periodic environmental time cue (Zeitgeber) only. However, the advantage of a circadian clock is that the daily rhythms are maintained in the absence of the Zeitgeber. So far, it has remained little investigated whether the circadian clock regulates emergence in solitary bees and if so, how daily rhythms of emergence are synchronized to their environment. Investigating the circadian clock and its Zeitgeber will help us to better understand how human impacts such as global warming or the management of bees in artificial nests for crop pollination influence the timing of the solitary bees and their synchronization with food plants, mating partners and competitors.

To date, the only evidence for a circadian clock involved in solitary bee emergence comes from the bee *Megachile rotundata*^7–9^. The species *M. rotundata* times its emergence to temperature rise and shows daily rhythms of emergence during exposure to daily temperature cycles^7^. Furthermore, it has been shown that a single, but strong temperature pulse can set the circadian clock in this solitary bee species and triggers daily rhythms of emergence lasting several days after the pulse^8^. Light was shown to be insufficient to trigger robust daily rhythms of emergence in *M. rotundata*^8,9^, although light can be an important Zeitgeber for daily rhythms in activity of honey bees and other insects, vertebrates and plants^4,10,11^ and emergence of flies and wasps^12–18^.

Which environmental cue is used as Zeitgeber may vary among clock-controlled behaviors, developmental stages and species. While the summer bee *M. rotundata* overwinters as pre-pupa, resumes pupal development in spring and emerges directly after eclosion from the pupa, solitary bees that emerge in spring eclose already in the autumn of the previous year. They enter winter diapause after adult eclosion^19^ and remain immobile inside their cocoons until emergence in spring^20^. The separation of emergence from eclosion by an interval of several months enables spring bees to emerge as soon as temperatures start rising in spring^21^. In contrast, *M. rotundata*, emerges shortly after eclosion^7–9^. To our knowledge, there have been no studies on the circadian timing of emergence in an insect species whose emergence is dissociated from eclosion by diapause.

In this study, we focused on the spring bee *Osmia bicornis* (“red mason bee”), which provides the possibility to study the timing of emergence which is dissociated from eclosion. *O. bicornis* occurs in wild but also in managed populations, which are used for crop pollination. The commercial value of solitary bees in pollination management has recently received increased attention and *O. bicornis* is a species highly promising in pollination services all over Europe^22–24^.

In our study, we tested the hypothesis that a circadian clock controls the emergence of *O. bicornis*, and that this results in the daily synchronization of all bees (males and females). Secondly, we hypothesized that light and temperature are Zeitgebers in *O. bicornis* and can cause daily rhythms of emergence, because both light and temperature have been found to set the circadian clock in several insects^10,25^. Furthermore we were interested in whether a Zeitgeber that proved to be not involved in the timing of emergence, can be involved in the timing of locomotion after emergence.

## Material

Cocoons of the red mason bee *Osmia bicornis* (Hymenoptera: Apiformes: Megachilidae) were purchased from a commercial supplier of solitary bees (WAB-Mauerbienenzucht, Konstanz, Germany) in October 2015, 2016 and 2018. Cocoons overwintered inside a climate chamber at 4 °C, 60% RH (relative humidity) and complete darkness. Temperature and humidity sensors (Driesen and Kern DK390 ECH20 HumiLog GP “rugged” and MSR Electronics GmbH, Seuzach, Switzerland) monitored the environmental conditions during the experiments.

## Experimental Design

### First emergence experiment

Due to limitations in our monitoring equipment we used two different approaches to monitor emergence events: the free running rhythm in emergence (i.e. the daily rhythm of emergence events in a population measured under constant conditions) was investigated with an infrared(IR)-beam based system while the effects of the Zeitgebers temperature and light on emergence rhythms were recorded via a camera monitoring system. The climate chambers used for the experiment were with temperature control and either a saltwater bath (saturated NaCl solution) at the bottom of the cabinet, buffering relative humidity at 71.21% (±8.58 SD) RH (Panasonic Cooled Incubator MIR-254-PE), or with an integrated humidity control set to 75% ± 10 SD) RH (Percival INTELLUS, CLF Plant Climatics GmbH, Wertingen, Germany). Before the start of the experiment the temperature was raised from storage conditions (4 °C) to emergence conditions (15–25 °C) during pre-emergence phase. This was done differently in the treatments according to the experimentally defined conditions (see for details Fig. S1).

#### IR-beam monitoring system

In this approach, we used a commercially available IR-beam based system (LAM16) by Trikinetics to assess emergence rhythms in constant conditions after synchronizing the bees’ clocks via daily temperature cycles. In this set up, 209 cocoons were placed individually in pyrex glass tubes (⦰16 mm) equipped with sugar syrup supplies (Apiinvert, Südzucker, Mannheim, Germany) and water *ad libitum* (see also^26^ for set up). Afterwards they were exposed to temperature cycles (temperature increment steps every 24 h: ΔT = 10 °C) for four days which stepwise raised the temperature in the incubator (Percival INTELLUS, CLF Plant Climatics GmbH, Wertingen, Germany) during pre-emergence phase to trigger synchronized emergence of the bees afterwards (Fig. S1). After four complete temperature cycles (12 h:12 h) the environmental conditions were kept constant (DD: constant darkness, 19.2 °C ± 0.1 °C SD, 75 ± 10% RH SD), which marked the beginning of the experiment (ZT = 0). An emergence event was defined as a bee’s crossing of the IR-beam during its first foraging activity, usually directly after the bee leaves the cocoon.

#### Camera monitoring system

We used the camera monitoring system to assess whether daily temperature and/or light-dark (LD) cycles can synchronize emergence behavior in *O. bicornis*. On the 7^th^ of April, 170 cocoons per treatment were individually placed in ID-labelled plastic tubes which were in turn sealed with cotton wool (Fig. S2/A,B). During pre-emergence phase (four days) temperature slowly increased from 4 °C to 20 °C, with four small temperature increment steps (∆T = 1 °C) per day randomly distributed across the day, to prevent the induction of daily rhythms of emergence (Fig. S1). On the first experimental day we established two different treatments in separate climate chambers (Panasonic Cooled Incubator MIR-254-PE). One climate chamber provided constant darkness and a temperature cycle (TC 12 h:12 h) of 12 hours high temperature (25 °C) and 12 hours low temperature (15 °C) (mean temperature ± SD: 19.91 °C ± 4.94). In the second climate chamber the temperature was set to 20 °C (mean temperature ± SD; 19.92 °C ± 0.07) and the LD cycle (LD 12 h:12 h) consisted of 12 hours of light (240 lux) and 12 hours of darkness (2 lux). To avoid disturbing their natural behavior, we left the pre-emerged adult bees in their cocoons but ensured that enough light could penetrate the cocoon shell. We measured the transmittance of empty cocoons with the help of a photometer (luxmeter MS-1300, Voltcraft, Hirschau, Germany) and found that, on average, 40 lux of the applied 240 lux reached the bee inside the cocoon. We attached cameras (Raspberry Pi Modell B, JOY-IT, Neukirchen-Vluyn, Germany) inside the chambers 12 cm above the cocoons (Fig. S2/A). These cameras captured one picture every 30 minutes. In these pictures, newly emerged bees were easily distinguishable from unopened cocoons. In order to obtain high-quality images in complete darkness we installed six infrared LEDs (SOLAROX LED Strip infrared, wavelength 940 nm, Solarox Holding Gmbh, Dessau-Roßlau, Germany) per camera. Infrared light cannot be detected by bees^27^ so the LEDs were kept switched on for the entire duration of the experiment. For the analysis in both approaches, we focused on the emergence data between the 11^th^ and the 24^th^ of April, as hardly any bees emerged before or after this time window.

### Second emergence experiment

We used the IR-beam monitoring system to investigate a possible sex difference in day time of emergence under temperature cycles and constant darkness. This experiment began on the 25^th^ of May 2017. We raised the temperature from storage conditions (4 °C) to 10 °C in one step before starting the experiment. In contrast to the first experiment, temperature during the second experiment was kept between 10 °C and 20 °C and temperature was increased and decreased gradually over 6 hours. We hypothesized that long temperature ramps would enable us finding even small differences between males and females regarding the time of day at which clustered emergence events occurred. The temperature was raised gradually from 10 °C to 20 °C in a 6 h lasting temperature ramp. The maximum temperature of 20 °C was hold 6 h and then temperature decreased gradually in a 6 h lasting ramp to 10 °C again. A period of 6 h at 10 °C followed afterwards. This whole cycle was repeated several times. Data were collected with a 1 minute resolution, in order to detect small differences in clustering of emergence events of males and females.

### Locomotion experiments

To test whether rhythms in locomotion of emerged adult solitary bees can be synchronized via light cycles, we introduced emerged bees to monitor tubes in the same IR-beam based activity monitoring system as the one used to measure emergence events with. The system picked up any movement of the bee detected by the IR-sensors. The bees had emerged a few days earlier in an emergence tent (made of gauze, size: 60 cm × 60 cm × 56 cm) with sugar syrup (Apiinvert, Südzucker, Mannheim, Germany) and water ad libitum (20 °C, RH 45%). In the IR-beam based activity monitoring system, the animals were then exposed to an LD cycle (12 h:12 h) for 3 days. The daytime of the first light signal did not coincide with the daytime the temperature had been raised from 4 °C to emergence conditions (20 °C) a few days earlier. Then 5–6 days of constant conditions (DD, constant temperature and humidity) followed. Afterwards, Bees were again submitted to an LD (12 h:12 h) cycle, with the same light intensity as the previous one but the light phase was shifted by 6 hours, for 3 days and then they were once again exposed to DD conditions. All environmental conditions except for light signals were kept constant during the experiment (19.7 °C ± 0.1 SD, RH 41% ± 0.7 SD) and activity signal acquisition was binned in 1 minute intervals. A total of 16 adult emerged bees were submitted to either 200–400 lux (N = 12) or 10–30 lux (N = 4) during the light phase in two separate experiments. We evaluated a possible activity shift due to synchronization by light cycles at least one time per individual. Nine individuals lived long enough for us to evaluate a second activity shift. We used white light LEDs emitting light with a spectrum of 440 nm – 750 nm wavelengths. This simulates the range of daylight spectrum visible for humans, but the emission peaks (at 620 nm and 450 nm) were slightly different from daylight spectrum.

### Statistical analysis

We removed bees from the climate chambers at the end of the experiment and determined their sex. We determined rhythmicity separately for the three treatments of the first emergence experiment in an autocorrelation analysis with a time resolution of 30 minutes (software R, version 3.0.2). We found the IR-beam set up approach to be highly comparable to the camera monitoring with this time resolution. Rhythmicity occurred if the data point distribution suggested a periodic occurrence of emergence events. The autocorrelation function analyzes the data for rhythmicity. It indicates the internal correlation between observations as a function of time lag between them. Rhythmicity in the data set is true, if the autocorrelation function peaks several times above the 10% confidence interval. Rhythmicity strength within the three treatments was identified by the rhythmicity index (RI) after implementing a butterworth filter on the data (matlab, version R2015b, developed by Joel Levine and method described in^28^). The RI represents the third peak in the smoothed autocorrelation function and defines the significance of the rhythm (RI > 0.3 = highly significant, 0.1 < RI < 0.3 = significant, RI < 0.1 = not significant). Additionally, we calculated the emergence period (P), i.e. period between clustered emergence events, via autocorrelation analysis and maximum entropy spectral analysis (MESA). Emergence and locomotion actograms were generated with the ImageJ plugin ActogramJ and FRPs (free running period of the endogenous clock measured in activity rhythms under constant conditions) of individual bees were calculated with the same plugin (Chi-square analysis, p = 0.05, smoothing factor 10)^29^. For calculation of mean emergence phase and further statistical analysis we used the software R (version 3.0.2). After testing for directedness of emergence events with the Kuiper’s test of uniformity, differences in mean distribution of emergence time points between the treatments were tested with Watson U^2^ test.

## Results

### Emergence experiments

**Figure 1:**
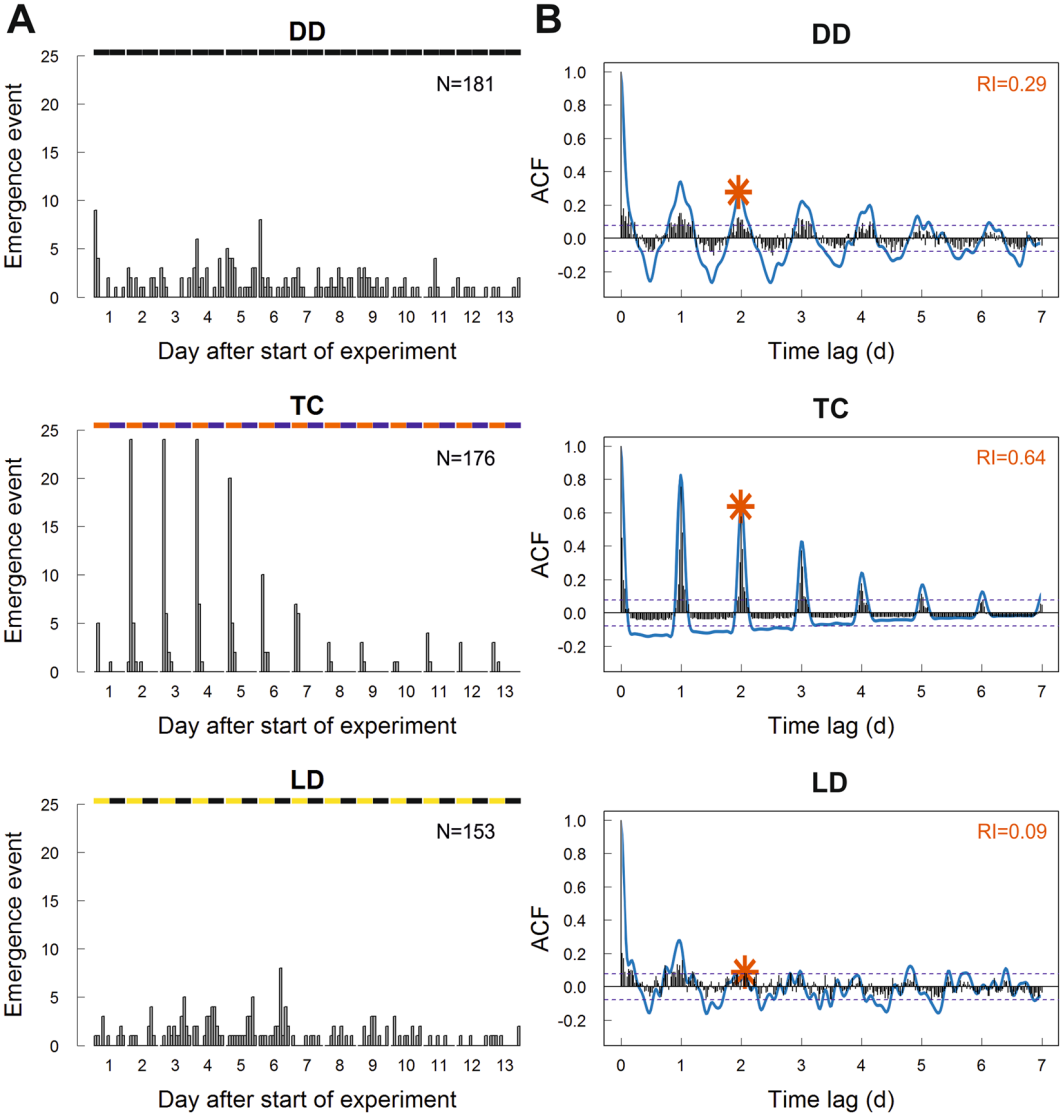
Analysis of rhythmicity in emergence under different environmental conditions. Separate graphs are shown for the treatments constant darkness (DD), temperature cycles (TC) and light-dark cycles (LD). Shown are the raw data of the emergence events throughout 13 days, plotted with a time bin of 2 hours (**A**), and the associated autocorrelation function (ACF) for the emergence events plotted with a time bin of 30 minutes (black bars), the 10% confidence interval (blue dotted line), the smoothed autocorrelation function (blue graph) and the rhythmicity index (RI, marked by the red asterisk) (**B**). The RI indicates rhythmicity strength (RI > 0.3 = highly significant, 0.1 < RI < 0.3 = significant, RI < 0.1 = not significant). X-axis shows the time lag between correlated emergence events. Daily rhythms of emergence are indicated by the autocorrelation function peaking above the confidence interval several times every approximately 24 h. Illumination bars on top of graphs in (**A)** depict the light regime in the different treatments (black: darkness, red: high temperature (25 °C), blue: low temperature (15 °C), yellow: light). Constant temperature in DD and LD, constant darkness in TC.

The raw data of the emergence events across 13 days, plotted with a time bin of 2 hours **(**Fig. 1A), demonstrate that under constant conditions (treatment DD) following synchronization with temperature cycles bees emerged with a daily rhythm. Under temperature cycles (treatment TC) bee emergence was tightly regulated by the Zeitgeber temperature, as most bees emerged within a 2 hour time frame every day, whereas under light-dark (LD) cycles (treatment LD) bees emerged with no clear rhythmicity. The associated autocorrelation functions (ACF) for the emergence events with a time bin of 30 minutes (Fig. 1B) strengthen these observations for all three treatments (DD, TC and LD). Comparing rhythmicity strength between treatments with the RI (Fig. 1B**/DD and TC**), the tight regulation of emergence by temperature cycles is mirrored in the highly significant rhythmicity (RI = 0.64) of bees emerging in TC, while the synchronized emergence without Zeitgebers in DD is slowly fading and the RI is lower (RI = 0.29) but still significant. On the other hand the bees emerging under LD cycles seem to be hardly synchronized and the RI is just below the significance level (RI = 0.09) (Fig. 1B**/LD**).

Bees showed an emergence period of 23.8 h under constant conditions (DD), while the emergence period under synchronizing temperature cycles (TC) was 24.0 h (Table 1).

**Table 1:** Rhythm analysis of emergence of *O. bicornis* under different environmental conditions (first emergence experiment).

Treatment	Emergence period (P) [h]	Rhythmicity index (RI)	Maximum entropy spectral analysis (MESA) [h]
DD total	23.8	0.29	24.1
DD male	23.8	0.24	23.9
DD female	22.8	0.22	23.6
TC total	24.0	0.64	23.8
TC male	24.0	0.56	24.5
TC female	24.0	0.58	24.8
LD total	24.8	0.09 (n.s.)	23.8
LD male	22.5	0.11	22.0
LD female	24.2	0.08 (n.s.)	25.1

Table shows emergence period (P) calculated in the autocorrelation function, rhythmicity index (RI) as measurement of rhythm strength and emergence period calculated with maximum entropy spectral analysis (MESA) for the whole population as well as male and female subpopulation. Treatments were conducted under different environmental conditions: DD (constant conditions), TC (temperature cycles of 12 h high and 12 h low temperature) and LD (cycles of 12 h light and 12 h darkness). RI > 0.3 = highly significant, 0.1 < RI < 0.3 = significant, RI < 0.1 = not significant.

**Figure 2:**
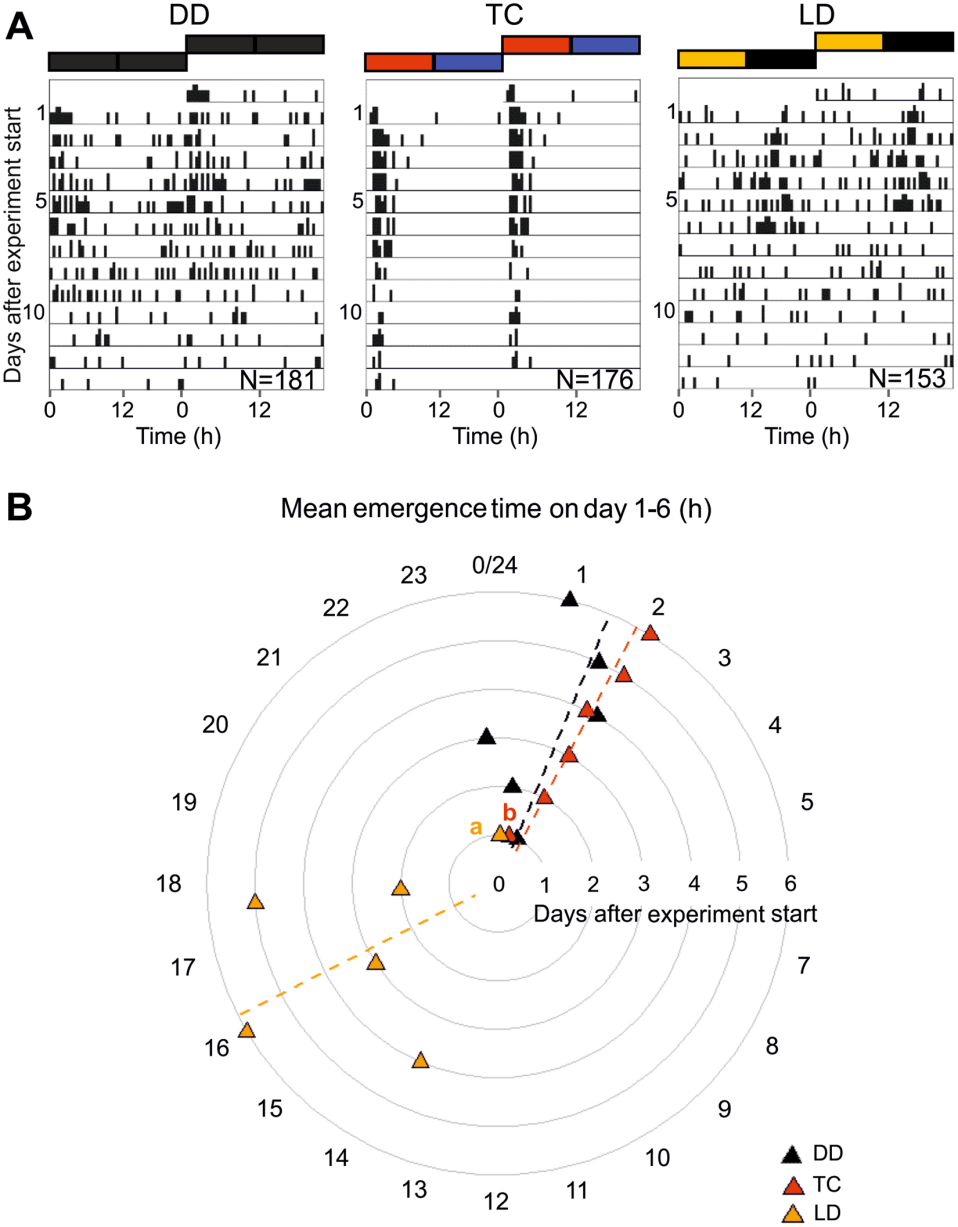
Comparison of emergence period and emergence activity patterns under different environmental conditions. (**A**) Double plotted emergence actograms of test bees in constant conditions (DD), with temperature cycles (TC) and light-dark cycles (LD). Emergence events in actograms are plotted in data bins of 30 minutes for the days 1 to 13 after experiment start and environmental bars depict the different conditions of the treatments (black/black: constant conditions; red/blue: 12 h high temperature/12 h low temperature; yellow/black: 12 h light/12 h darkness). (**B)** shows the mean phases of emergence events on day 1–6 (triangles) and the mean emergence phase over all six days (dotted lines) for the different treatments (DD, TC, LD), which indicate a differential phase relationship for LD compared to the other treatments. Circular axis: “time of the day” after experiment onset. Watson U^2^ test provides significant difference in emergence phase distribution on day 1 between treatments TC and LD.

To reveal the time of day at which emergence occurred, we plotted the data as actograms (Fig. 2A). In the actograms, an accumulation of emergence events at a certain time of day was observable under all three conditions, even though the autocorrelation analysis revealed no significant rhythm under LD conditions. However, the time of day at which emergence mainly occurred was clearly different under LD (Fig. 2A**/LD**) as compared to TC and DD (Fig. 2A**/TC and DD**). Under TC and DD (Fig. 2A**/TC and DD**), most bees emerged at the beginning of the day (~2 hours after the daily temperature increase in TC or ~2 hours after the previous temperature increase in DD). Under LD conditions, the bees appeared to emerge in the middle of the night (Fig. 2A**/LD**). From day 7 onwards we were no longer able to visually detect rhythmicity in the emergence actograms in DD and LD so we only calculated mean emergence time points from day 1 to day 6 and plotted the results for all three conditions into one phase plot (Fig. 2B). In treatment TC, mean emergence was tightly regulated by the Zeitgeber temperature as most of the bees emerged at Zeitgeber time (ZT) 2 (equals 2 hours after beginning of the day). In DD, the clocks of the bees had been synchronized by a temperature cycle with the same phase relationship like in treatment TC and the bees emerged at a similar time point like the bees in TC. As expected for bees in constant conditions, emergence time points showed increasing divergence over the days because of individual FRPs. The phase of mean emergence hence varied more over the days in DD (from 0–2 h of the day) than in TC (at 2 h of the day). The phase of mean emergence in LD also highly varied between day 1 and 6 (from 14 to 24 hours after beginning of the day). The difference in phase relationship to the treatments DD and TC is particularly striking: mean emergence over the different days in LD occurs 16 hours after lights-on, which corresponds to the middle of the night, whereas for DD and TC it lies between 1.5 and 2 hours after the beginning of the day. Even on day one, which shows the smallest difference in phase relationship among treatments, distribution of emergence phases of the two treatments with Zeitgebers temperature and light were significantly different (Watson U^2^ test:0.1899, p < 0.05). The phase of emergence in LD correlates with the time at which the last temperature increment step occurred during the pre-emergence phase. This implicates that synchronization of emergence in LD was caused by this small increase in temperature (∆T = 1 °C) and not by light signals.

The analysis of daily rhythms of emergence for the male and female subpopulations produced highly similar results. Rhythmicity in emergence was alike for males and females in all three treatments with a slight tendency of a stronger rhythmicity in the male subpopulation under DD and LD conditions (Table 1). The analysis furthermore revealed an emergence period of 23.8 h for the male subpopulation and 22.8 h for the female subpopulation under constant conditions (DD) and 24.0 h for both the male and the female subpopulation under synchronizing temperature cycles (TC).

**Figure 3:**
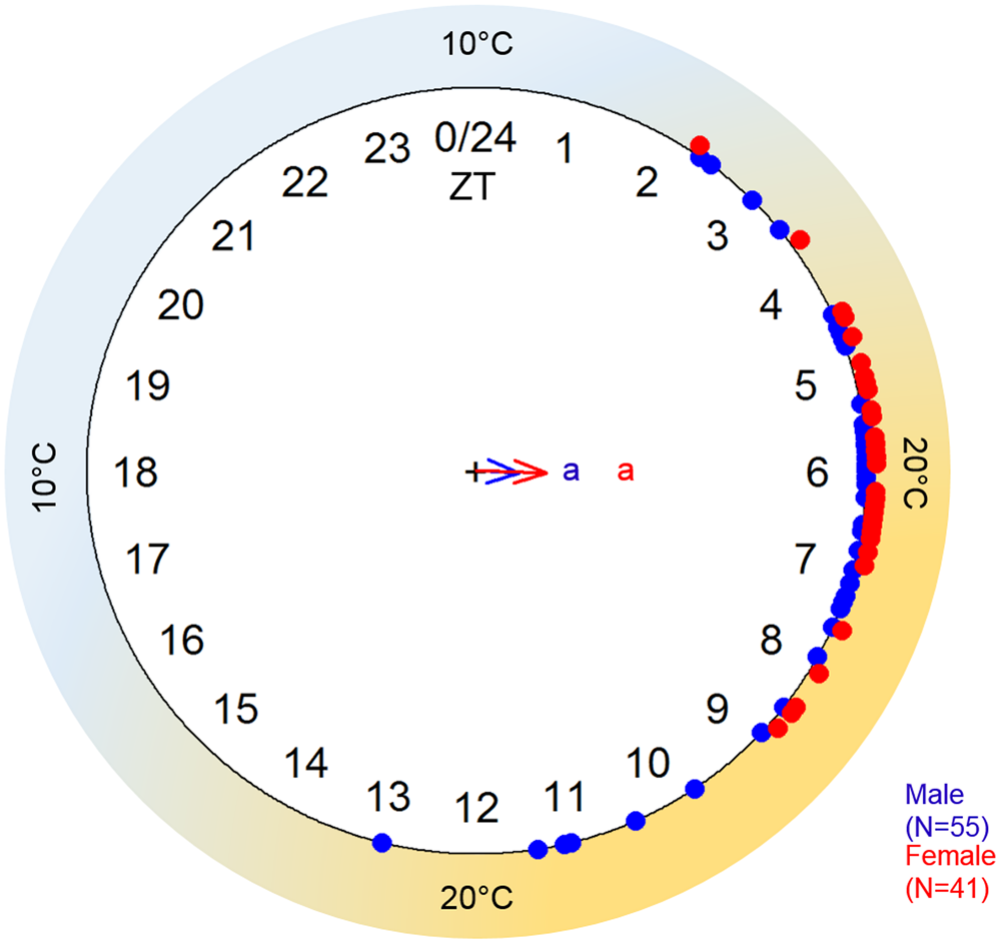
Emergence of male and female O. bicornis under temperature cycles with ramping temperature adjustment. Circular 24 h plot of emergence phases of male (blue dots) and female bees (red dots) (1 min time resolution). Bees emerged during several days under daily temperature cycles with slowly adjusting temperature conditions (hour ZT 0–6: temperature ramp from 10 °C to 20 °C; hour ZT 6–12: 20 °C; hour ZT 12–18: temperature ramp from 20 °C to 10 °C; hour ZT 18–24: 10 °C). We found no difference in distribution of male and female emergence phases (Watson U^2^ test: p > 0.05). The best-suited emergence temperature seems to be approximately 20 °C for both sexes (red and blue arrows indicate the mean emergence phase averaged over all days for female and male bees).

Emergence phases in treatment TC did not differ for males and females (Watson U^2^ test: p > 0.05). This was also the case for emergence in the second emergence experiment with daily slowly increasing and decreasing temperature (Watson U^2^ test: p > 0.05, Fig. 3). The long temperature ramps would enable us finding even small differences in the time of day at which males and females mainly emerged respectively. Nevertheless, we did not see differences of this type.

Analysis of emergence period with MESA (maximum entropy spectral analysis) (Table 1), a highly sensitive method for detecting periodicities^30^, confirmed trends in our autocorrelation analysis results, with the sole exception that emergence period calculated with MESA marginally differed for the different subpopulations in the TC treatment. In this case we rely on autocorrelation analysis because MESA does not provide information on the significance of the calculated circadian emergence period.

### Locomotion experiments

**Figure 4:**
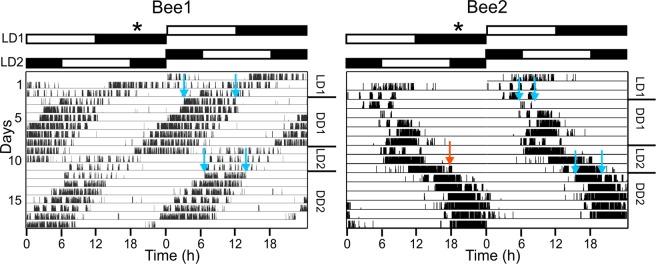
Synchronization of rhythms in locomotion of emerged *O. bicornis* via light cycles. Two representative actograms (activity signals plotted in double plots) for synchronization of locomotion rhythms of emerged bees via light cycles (LD cycles). Bee1 was subjected to a light intensity of 200–400 lux and Bee2 to a light intensity of 10–30 lux during LD cycles (LD1 and LD2). The LD cycles are followed by several days in constant conditions (DD1 and DD2) which are indicated in the right margin. Bee1 was synchronized with LD1 after 3 days. On the first day of constant darkness (DD) the activity phase of the bee (marked by two blue arrows) is at the time of the day when the previous light phase of LD1 was and here the free running rhythm of the bee also starts. This demonstrates the setting of the circadian clock by daily light cycles. Under the second LD cycle (LD2) Bee1 shifted its activity rhythm even faster to synchronize with the light cycle and the activity phase of the first day in constant conditions (marked by a second set of blue arrows) coincides with the light phase of LD2. The clock of Bee2 was also set by the light cues. Bee2 adjusted its activity to the light phase in LD1 and later in LD2 respectively. It exhibited its main activity on the first day in DD1 (marked by two blue arrows) and in DD2 (marked by a second set of blue arrows) at the time of the day at which the light phase had been in the preceding LD regime. In general, bees, which were subjected to a lower light intensity, preferred to be active close to the lights off signal and showed some activity during the early dark phase (red arrow in the right actogram). Environmental bars on top depict the light regimes in LD1 and LD2 (white: light phase; black: dark phase). The approximate time of the day at which bees were moved from storage conditions (4 °C) to experimental conditions (20 °C) (before they emerged) is marked by an asterisk in the environmental bar panel.

By exposing emerged adult solitary bees into two differently phased light regimes with a period of constant conditions (DD) in between and afterwards, we tested if rhythms in locomotion of the emerged bee could be synchronized by light cycles. We inspected the actograms of the individual bees and found that animals always displayed their main activity phase on the first DD day at the time of the day the light phase had been in the previous LD cycle (Fig. 4). This demonstrates that light cycles can set the clock to synchronize daily rhythms in locomotion.

The FRPs of the individual solitary bees shown in the locomotion actograms under DD are, on average, less than 24 h (23.35 h ± 0.34 h SEM), which fits to studies in honey bees^11,31,32^. Interestingly, the FRPs of *O. bicornis* show a high inter individual variation with periods ranging from 20.07 h to 26.77 h. A similarly high variation in inter individual FRPs has been reported for a honey bee hybrid in a study collecting locomotion data from individuals of different colonies^31^. It has been shown that the FRP depends on different factors like age, genetic background and individual state^33–35^. Therefore, the high inter individual variation we found may be due to the diverse genetic background of the solitary *Osmia* bees, which were collected by the bee keeper with trap nests at different locations. We neither controlled for the exact age (the bees emerged in the course of a few days) nor did we analyze males and females separately. These could be additional reasons for such high variation in FRPs.

## Discussion

We demonstrated that emergence of *O. bicornis*, a bee species that overwinters as adult within its cocoon, is governed by the circadian clock and temperature cycles synchronize the clocks for emergence rhythms. We hence describe for the first time daily rhythms of emergence of a species which shows emergence dissociated from eclosion by diapause. This demonstrates that emergence *per se* is clock regulated in this bee and the timing of emergence to a specific time of the day is not influenced by the eclosion process. We showed that, synchronized emergence mainly depended on temperature signals and light played an evanescent role for daily rhythms of emergence. In contrast, daily rhythms in locomotion of emerged adult bees were synchronized after exposure to LD cycles. We also found that male and female bees equally favor “morning hours”, after temperatures started to rise, to emerge.

### High temperature and low light sensitivity of the circadian system synchronizing emergence is conserved in different bee species

In *O. bicornis*, emergence is separated from eclosion by several months of diapause, which makes this species perfect for investigating daily rhythms of emergence. So far, most reports on daily rhythms of emergence concern species whose emergence behavior is closely associated with eclosion. It has been equally shown for various drosophilid species as well as the onion fly, *Delia antiqua*, and the flesh fly, *Sarcophaga crassipalpis*, that both light and temperature cycles can trigger daily eclosion rhythms and associated emergence rhythms^12–14,36–40^. However, under natural conditions, the insect is simultaneously submitted to several Zeitgebers and in differently phased temperature and LD cycles, temperature was shown to be the synchronizing factor of eclosion events in the tsetse fly (*Glossina morsitans*) and the flesh fly (*Sarcophaga crassipalpis*)^38,41^. The potency of temperature synchronization in eclosion may vary with the phase angle between the two Zeitgeber cycles and temperature amplitude in the Zeitgeber signal as demonstrated for the onion fly and *Drosophila pseudoobscura*^14,40^. Eclosion and associated emergence in *D. melanogaster* is controlled via interaction of the central clock residing in the brain with the peripheral clock in the prothoracic gland, where the production of ecdysone is controlled^42–45^. However in several hymenoptera, an interval of hours to days lies between eclosion and emergence and also here it has been demonstrated that light and/or temperature cycles cause daily rhythms of emergence, but the molecular basis of the circadian clock influencing emergence of these animals is unknown^7,8,15–18^. Solitary bees represent an exception from the rule, because they are the only insects of the above mentioned, in which light signals have been insufficient to trigger robust daily rhythms of emergence as shown for *M. rotundata*^8,9^.

Accordingly, emergence events of *O. bicornis* were not synchronized to the ongoing LD regime in our study. Nevertheless, we observed a daily rhythm in emergence with accumulation of emergence events at a day phase coinciding with the day phase of the last temperature step 11 hours before the beginning of the experiment (∆T = 1 °C; see Fig. S1 for time of the last step in raising the temperature from storage to emergence conditions). As we kept all other environmental factors in the incubator constant, this 1 °C increase may be the cause of the slight synchronization of emergence events in the LD treatment. Similarly, temperature changes with very small amplitudes have been reported to trigger daily rhythms of emergence in *M. rotundata* (∆T = 2 °C; 0.33 °C/h in a 12 h temperature ramp)^7,9^ and the tsetse fly (∆T = 0.4 °C)^41^. In line with our observations, Tweedy and Stephen (1970)^8^ report that *M. rotundata* bees from populations under daily LD regime emerged in an unsynchronized fashion unless they were subjected to a low temperature pulse. The observed high temperature response and low light sensitivity of the circadian clock in the synchronization of emergence seems to be conserved among different solitary bee species. Even in an experiment with very high light intensities (approximately 6000 lux) only a mild synchronization of emergence events was found for *M. rotundata* under LD and the rhythms were not sustained in a competition situation with differently phased temperature cycles^9^. We cannot exclude that *O. bicornis* might show some synchronization reaction in LD cycles with similarly high light intensity, but Bennett and co-authors (2018)^9^ also conclude that even light signals with very high intensities play an evanescent role for setting the clock for daily rhythms of emergence in *M. rotundata*, compared to temperature signals. Future studies including experiments with competing temperature- and light-dark-cycles may reveal further details on the role of temperature and light in daily rhythms of emergence of *O. bicornis*.

### A discrete clock mechanism governs emergence in solitary bees

The pre-emergent adult phase in *M. rotundata* lasts only a few days^7,9^, while in *O. bicornis*, emergence behavior is completely decoupled from eclosion by winter diapause and here we show that emergence behavior *per se* is regulated by the circadian clock. Because robust daily rhythms of emergence were only triggered by temperature but not LD cycles in both bee types^8,9^, we assume synchronization of emergence may be controlled similarly in adult and pre-pupal diapausing bees. A temperature threshold for receiving the Zeitgeber signal or triggering the reaction may be another feature of the solitary bee emergence behavior, because we observed adjustment of the day phase of emergence in the second emergence experiment with gradually increasing temperature, so that bees emerged at approximately 20 °C.

As emergence is basically the onset of locomotion it may relate to this behavior in the bee. Nevertheless, activity rhythms in adult honey bees can only be set, if temperature amplitude between low and high temperature phase in daily temperature cycles is 7–10 °C or more, while even extremely low light intensities showed an effect on the circadian clock^4,46,47^. In locomotion experiments with two differently phased daily LD regimes followed by constant dark conditions, we show that synchronization of rhythms in locomotion by light cycles is also easily achieved in emerged adult solitary bees (*O. bicornis*). Emergence behavior on the contrary, was not affected by LD cycles, although illumination intensity in our experiments focusing on bees inside the cocoons and on emerged bees were within the range of light responsiveness of the circadian clock of adult mammals, honey bees and fruit flies (ranging from several hundred lux to less than 1 lux)^47–50^. This suggests a different regulation of synchronization of emergence and post-emergence locomotion by the circadian system. Light-susceptibility may increase with development from pre-emerged to emerged solitary bee. Similarly, in pupa of the onion fly, *D. antiqua*, an age dependent increase in sensitivity to daily LD cycles was observed in eclosion behavior essays^51^, while for example, flesh flies (*Sarcophaga argyrostoma*) and blow flies (*Lucilia cuprina*) displayed higher sensitivity to eclosion synchronizing LD cycles in early larval stages^52,53^. Future studies may reveal which physiological and/or molecular regulation factors play a role in the timing of emergence of solitary bees.

### Temperature seems the more reliable Zeitgeber for emergence of solitary bees

Temperature signals may be the more reliable Zeitgeber for pre-emerged, cavity-nesting bees like *O. bicornis* and *M. rotundata*, because they easily reach the bees in their nests. The importance of daily fluctuating temperature regimes for survival and developmental rate of solitary bees has been shown in earlier studies^54–56^. In our study, we were in fact unable to identify light as a potential Zeitgeber for setting daily rhythms in emergence, but a very small change in temperature could synchronize emergence events. We therefore conclude that light has evanescent importance in clock regulated synchronization of emergence, but temperature signals are extremely important for timing emergence of *O. bicornis*.

The bees in our experiments emerged during the “morning hours”, 2 h after temperatures started to rise, which enables them to maximize the time of their first day out of the cocoon for performing activities like mating, foraging and nest building. In addition, they seem to adjust emergence to times of the day at which temperatures are within a preferred temperature range, which we could observe in our experiment with ramping temperature. Here most bees emerged in a warm-temperature time window (approximately 20 °C). The phase relationship to the Zeitgeber cycle differed between emergence of bees in temperature cycles with strong temperature steps (at ZT 2, red dotted line in Fig. 2) and emergence of bees in temperature cycles with gradually ramping temperature (at ZT 6, blue and red arrow in Fig. 3). Under natural conditions, the temperatures would similarly not rise in a strong temperature step, but rather gradually. Waiting for pleasant day temperatures would ensure that the bees do not emerge under unsuited or even fatal environmental conditions. Furthermore, this highly temperature sensitive system suggests susceptibility to temperature changes caused by climate change. Nevertheless, we do not know if the higher temperatures alone may cause emergence behavior before the morning hours, which would go along with fitness costs to the bees. How the mechanisms of adjusting emergence to specific times of the day works and influences bee fitness remains to be investigated.

### Bees benefit from daily synchronized emergence of sexes

We did not find sex differences in the daily timing of emergence in solitary bees. The marginal differences in rhythmicity strength might simply be due to the fact that females emerged a few days later than males and, therefore, the last time signal they experienced lay further in the past (Table S1). The fact that rhythmicity strength of emergence in female and male subpopulation was equal under synchronizing daily temperature cycles confirms this assumption. In the wild, male *O. bicornis* emerge a few days earlier than females and wait for the females at the nest site, which ensures their reproductive success^21,57^. Even though male emergence starts several days before female emergence, the emergence periods of males and females strongly overlap. In a field experiment, about 50% of females were found to emerge during the emergence period of the males, and about 50% of males emerged during the emergence period of the females^58^. Thus, synchronisation of emergence of males and females to a specific time of the day is probably relevant under field conditions. *Osmia* females do not need assistance during emergence, unlike females of some *Clunio* species, which have been reported to be helped out of their pupal skin by the already emerged males^59^. We accordingly observed that male and female solitary bees emerged at the same time of the day, in the “morning hours” after temperatures began to rise. This is consistent with studies in *M. rotundata*^7^, but Yocum and co-authors (2016) did not discriminate between sexes. The fact that males and females show synchronized emergence in the morning may improve mating success for both sexes. Females may benefit from a synchronized emergence of males, because the chance to mate with “the best male” may increase when many males are present at the same time. Males also displayed synchronized emergence with a daily pattern, but emerged on average a few days earlier than the females. By doing this the males probably ensure to emerge before or at least at the same time as the females, which provides attractive males the possibility to mate with numerous females. Therefore, the synchronized presence of males may be facilitated by the synchronized emergence of females.

In summary, our study indicates a discrete circadian clock mechanism, which governs emergence in solitary bees decoupled from eclosion. High sensitivity to daily changes in temperature and low sensitivity to daily changes in light intensity in the regulation of synchronized emergence, as we found in *O. bicornis*, may be an adaptation of the clock to the cavity-nesting life style of solitary bees. Furthermore, the circadian system in solitary bees shows increasing responsiveness to light signals during adult development as shown in the locomotion rhythms of emerged bees. Finally, male and female *O. bicornis* bees show synchronized emergence behavior in the “morning hours” after temperatures started to rise. Our study poses a new insight into clock evolution in bees and delivers prospects for future functional studies on the circadian clock of solitary bees.

## Supplementary information

Supplementary information

Supplementary dataset 1–4
