# Supplementary material for: The circadian clock uses different environmental time cues to synchronize emergence and locomotion of the solitary bee Osmia bicornis
Source: Sci Rep. 2019 Nov 28;9:17748. doi: 10.1038/s41598-019-54111-3 (PMC6883065; doi:10.1038/s41598-019-54111-3)
Supplement: Supplementary file 1 — Supplementary information [file 41598_2019_54111_MOESM1_ESM.docx]

**Supplementary information**

**The circadian clock uses different environmental time cues to synchronize emergence and locomotion of the solitary bee *Osmia bicornis*.**

Katharina Beer, Mariela Schenk, Charlotte Helfrich-Förster, Andrea Holzschuh

**
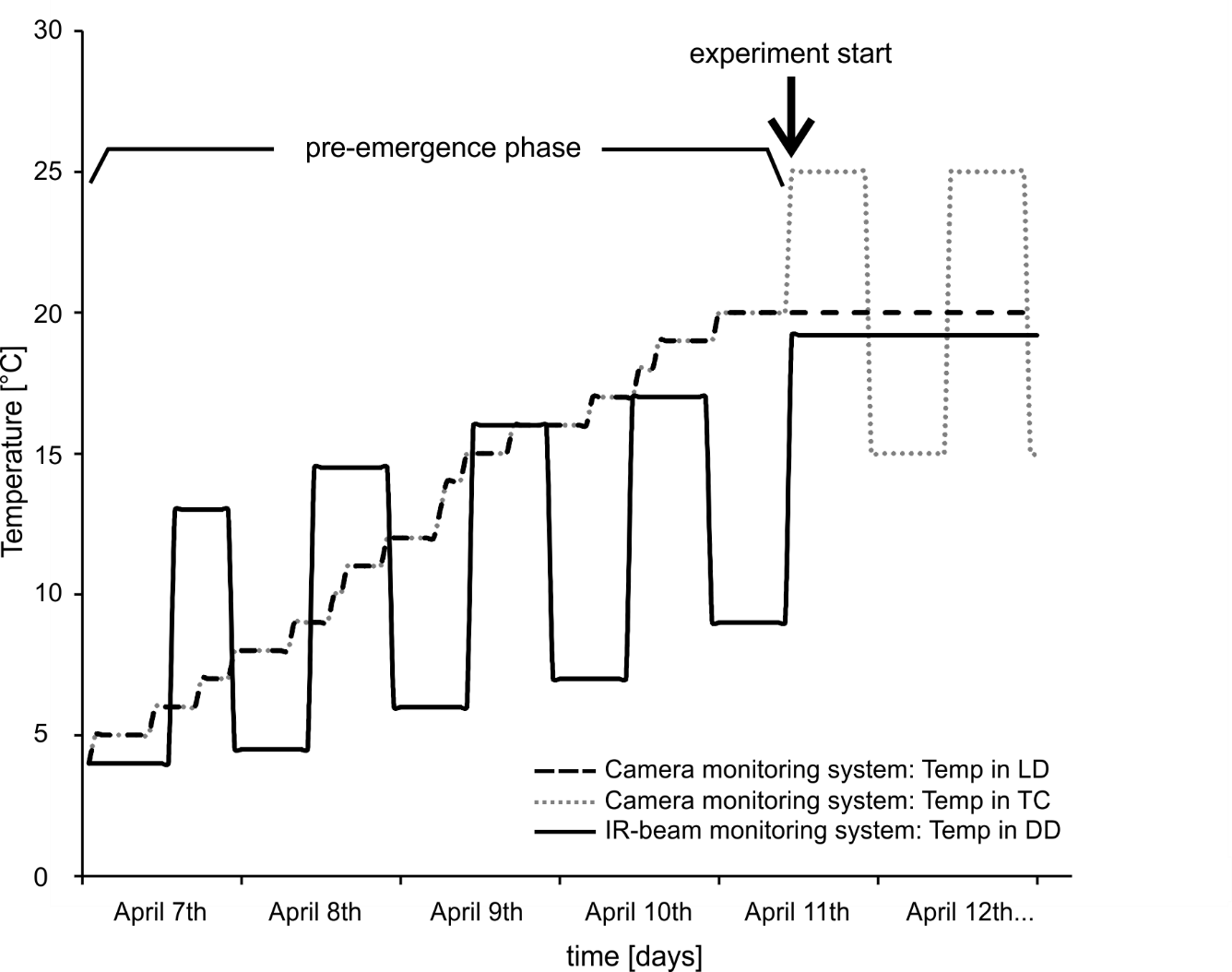
**

**Supplemental figure S1: Temperature treatments in first emergence experiment from storage conditions (4°C) to experimental conditions.** In all treatments, temperatures had to be adjusted to suitable emergence conditions (experimental conditions). This was done during the pre-emergence phase. Given we wanted to either synchronize the circadian clock of the bees or not do so, we adjusted temperatures differently in the different treatments. In the treatments with camera monitoring, i.e. treatments TC (temperature cycles) and LD (light-dark cycles), we wanted to avoid synchronization. We therefore used small irregular steps scattered throughout the day. In the treatment with IR-beam monitoring, i.e. treatment DD (constant environmental conditions), we synchronized the bees’ clocks with 4 cycles of high and low temperatures (12h:12h) before releasing them into constant high temperatures at experiment start ZT 0 (10:00, 11^th^ of April). The experimental temperature conditions, in which the bees emerged, were established from experiment start on. In the figure only one full day (April 12^th^) in experimental conditions is shown, but the same conditions persisted until the end of the experiment. These were constant 20°C (in LD), daily cycles of high and low temperatures (12h:12h) (in TC) and constant 19.2°C (in DD). There was additionally a daily light-dark cycle (12h:12h) in the LD treatment (not indicated in the figure).

B

A


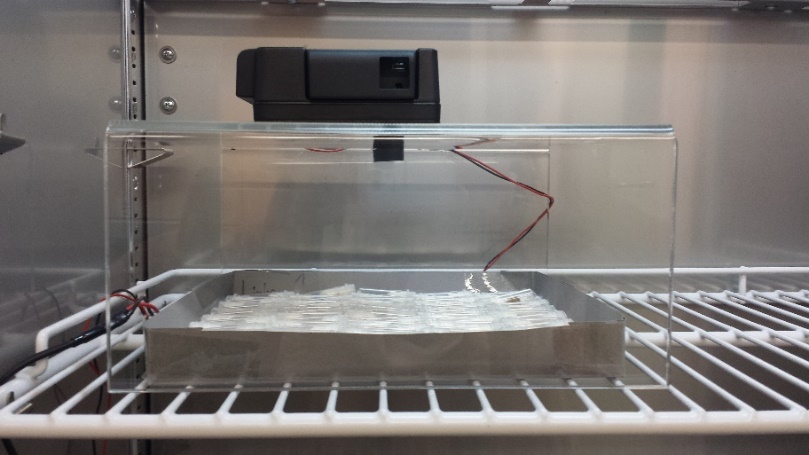

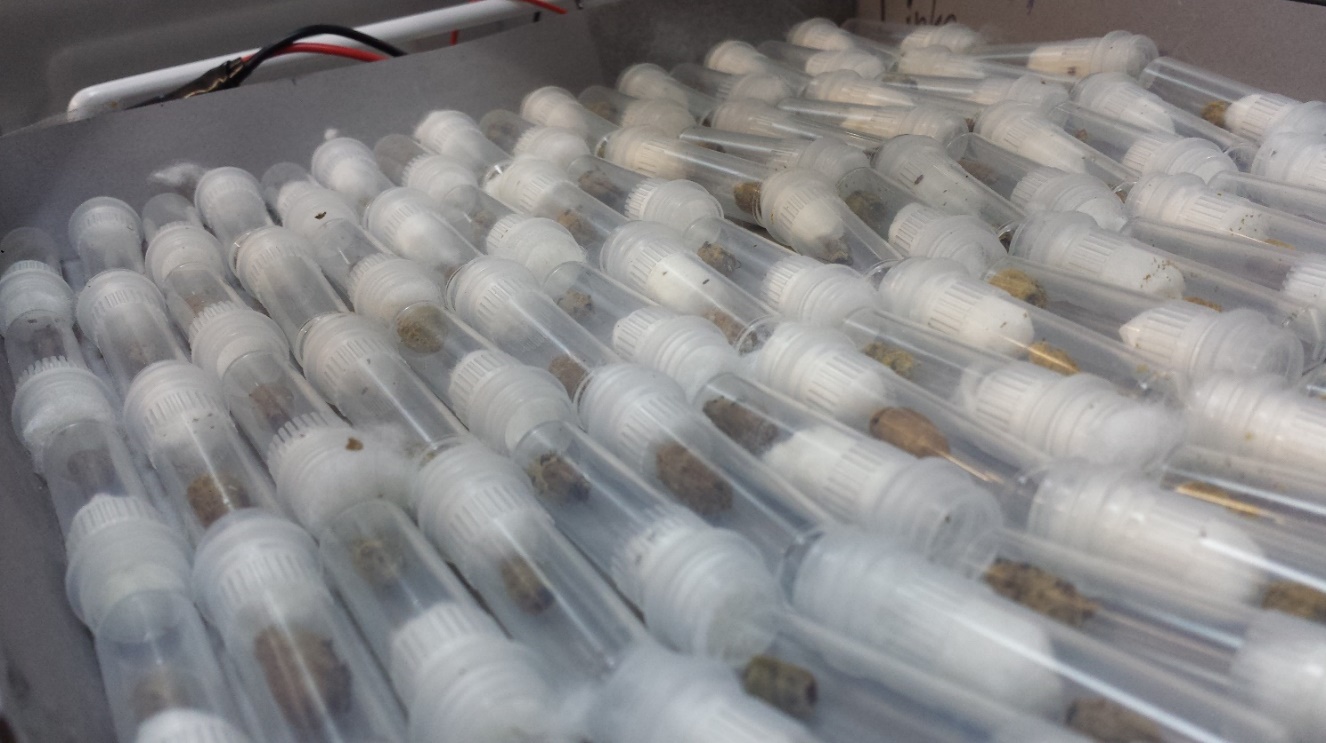


camera

cocoons

Infrared light

**Supplemental figure S2: Set up for monitoring emergence of the solitary bees via camera.** **A**: Picture of positions of camera, red light and cocoons in the incubator. **B**: Close up picture of cocoons individually enclosed in transparent tubes, sealed with cotton plugs. Camera recorded pictures every 30 minutes and infrared light was installed to enable picture taking during dark phases.

**Table S1: Statistics for emergence day of *O. bicornis*.** Males emerged a few days earlier than females in all treatments (DD: constant conditions, TC: temperature cycles, LD: light-dark cycles).

|  | **DD** | **TC** | **LD** |
| --- | --- | --- | --- |
| **Mean emergence day of males** | 4.0 ± 0.3  N=92 | 3.5 ± 0.2  N=103 | 3.4 ± 0.2  N=62 |
| **Mean emergence day of females** | 8.0 ± 0.3  N=89 | 6.4 ± 0.4  N=73 | 7.3 ± 0.3  N=91 |
| **p-values (Wilcoxon rank sum test)** | p<0.01 | p<0.01 | p<0.01 |
|  |  |  |  |
